# Supplementary material for: Dcifer: an IBD-based method to calculate genetic distance between polyclonal infections
Source: Genetics. 2022 Aug 24;222(2):iyac126. doi: 10.1093/genetics/iyac126 (PMC9526043; doi:10.1093/genetics/iyac126)
Supplement: iyac126_Supplemental_Material_File_1 [file iyac126_supplemental_material_file_1.pdf]

Supplementary Materials

S.1 A note on model assumptions

The assumption of independence of all interhost IBD variables at a given locus, while convenient, does not at first appear fully aligned with the goal of capturing interhost relatedness, as it leaves out a potential source of relatedness that could be present in the dependent case. For example, r_{x_1,y_1} and r_{x_2,y_1} could be both positive and still satisfy a "no intrahost relatedness" assumption ($r_{x_1,x_2} = 0$) with a certain type of dependence between $IBD_{x_1,y_1,t}$ and $IBD_{x_2,y_1,t}$. For a simplest special case of such dependence, consider the first two strains in the first sample being the unrelated parents of the first strain in the second sample; then

$$IBD_{x_2,y_1,t} = \begin{cases} 0 & \text{if } IBD_{x_1,y_1,t} = 1 \\ 1 & \text{if } IBD_{x_1,y_1,t} = 0 \end{cases}$$

The general case can be described as multiple strains in one infection (say, strains $i = 1, \ldots, m$) being unrelated to each other while all related to a strain in another infection (strain j). In that case, the sum $\sum_{i=1}^{m} IBD_{x_i,y_j,t}$ of corresponding IBD variables does not exceed 1: $\sum_{i=1}^{m} IBD_{x_i,y_j,t} \in \{0, 1\}$, and the working model will still be able to capture $\sum_{i=1}^{m} r_{x_i,y_j}$ by treating $\left(\sum_{i=1}^{m} IBD_{x_i,y_j,t}\right)$ as a single binary variable and thus arriving at the desired estimate of overall relatedness or individual relatedness estimates within the framework.

S.2 Constructing the likelihood

We start with the simplest case of $M = 1$, $\boldsymbol{r} = r$. Then

$$\begin{aligned} L(r; \boldsymbol{u_x}, \boldsymbol{u_y}, n_x, n_y, \boldsymbol{\pi}) &= \prod_{t=1}^{T} \sum_{s_x \in Q_{x,t}} \sum_{s_y \in Q_{y,t}} P\Big(S_{x,t} = s_x, S_{y,t} = s_y) \\ &= \prod_{t=1}^{T} \sum_{s_x \in Q_{x,t}} \sum_{s_y \in Q_{y,t}} \Big[P(S_{x,t} = s_x, S_{y,t} = s_x \,|\, IBD_{1,t} = 1)\, P(IBD_{1,t} = 1) \\ &\qquad + P(S_{x,t} = s_x, S_{y,t} = s_y \,|\, IBD_{1,t} = 0)\, P(IBD_{1,t} = 0)\Big] \\ &= \prod_{t=1}^{T} \sum_{s_x \in Q_{x,t}} \sum_{s_y \in Q_{y,t}} \Big[rP(S_{x,t} = s_x, S_{y,t} = s_y \,|\, IBD_{1,t} = 1) \\ &\qquad + (1 - r)P(S_{x,t} = s_x)P(S_{y,t} = s_y)\Big] \end{aligned}$$

If $IBD_{1,t} = 0$, $S_{x,t}$ and $S_{y,t}$ are independent, and any matching of the alleles between them occurred by chance. Using multinomial distributions, $P(S_{x,t} = s_x)\, P(S_{y,t} = s_y) = g\big(s_x; n_x, (\pi_t)\big)\, g\big(s_y; n_y, (\pi_t)\big)$. If $IBD_{1,t} = 1$, one of the shared alleles $a_{t,i} \in u_{xy,t}$, where $u_{xy,t} = u_{x,t} \cap u_{y,t}$, is matching because of

shared ancestry (if there are no shared alleles, $IBD_{1,t}$ cannot be 1). Fixing this allele in place and allowing other alleles in s_x and s_y to "fill" the rest of the haplotypes, we get

$$P\big(S_{x,t} = s_x, S_{y,t} = s_y \,|\, IBD_{1,t} = 1\big) = \begin{cases} 0 & \text{if } u_{xy,t} = \varnothing \\ \displaystyle\sum_{i:\, a_{t,i} \in u_{xy,t}} \pi_{t,i}\, g\big(s_x \backslash \{a_{t,i}\}; n_x - 1, (\pi_t)\big) & \\ \qquad \times g\big(s_y \backslash \{a_{t,i}\}; n_y - 1, (\pi_t)\big) & \text{otherwise} \end{cases} \tag{S.1}$$

When $1 < M \leqslant |s_{xy,t}|$ and $\sum_{i=1}^{M} IBD_{i,t} = m > 0$, the "fixed" portion of the interhost strain pairs can itself be thought of in terms of a multinomial distribution $Multinom(m, \pi_{t,1}, \ldots, \pi_{t,K_t})$, extending the base $M = 1$ case. We illustrate the process with $M = 2$:

$$\begin{aligned} P(S_{x,t} = s_x, S_{y,t} = s_y) = \quad & P(S_{x,t} = s_x, S_{y,t} = s_y \,|\, IBD_{1,t} = 1, IBD_{2,t} = 1)\, r_1 r_2 \\ & + P(S_{x,t} = s_x, S_{y,t} = s_y \,|\, IBD_{1,t} = 1, IBD_{2,t} = 0)\, r_1(1 - r_2) \\ & + P(S_{x,t} = s_x, S_{y,t} = s_y \,|\, IBD_{1,t} = 0, IBD_{2,t} = 1)\, (1 - r_1) r_2 \\ & + P(S_{x,t} = s_x, S_{y,t} = s_y \,|\, IBD_{1,t} = 0, IBD_{2,t} = 0)\, (1 - r_1)(1 - r_2) \end{aligned}$$

$P(S_{x,t} = s_x, S_{y,t} = s_y \,|\, IBD_{1,t} + IBD_{2,t} = 1)$ is equal to (S.1) and

$$P(S_{x,t} = s_x, S_{y,t} = s_y \,|\, IBD_{1,t} = 1, IBD_{2,t} = 1) = \begin{cases} 0 & \text{if } |s_{xy}| < 2 \\ \displaystyle\sum_{s^{(2)} \subseteq s_{xy}} g\big(s^{(2)}; 2, (\pi_t)\big) & \\ \qquad \times g\big(s_x \backslash s^{(2)}; n_x - 2, (\pi_t)\big) & \\ \qquad \times g\big(s_y \backslash s^{(2)}; n_y - 2, (\pi_t)\big) & \text{otherwise} \end{cases}$$

Conditional probability $P(S_{x,t}, S_{y,t} \,|\, IBD_{1,t}, \ldots, IBD_{M,t})$ is the same for all $(IBD_{i,t})_{i=1}^{M}$ sequences with the same $\sum_{i=1}^{M} IBD_{i,t}$, which makes calculations considerably faster.

S.3 Breaking down the log-likelihood function

To get some intuition on the behavior of the log-likelihood function and how it is affected by the sample data, COI, and population allele frequencies, we explore the likelihood for a single locus (sample size is 1) when $M = 1$ ($\boldsymbol{r} = r$). For locus t,

$$\begin{aligned} L(r; u_{x,t}, u_{y,t}, n_x, n_y, \boldsymbol{\pi}) = \quad & \sum_{s_x \in Q_{x,t}} \sum_{s_y \in Q_{y,t}} r P(S_{x,t} = s_x, S_{y,t} = s_y \,|\, IBD_{1,t} = 1) \\ & + \sum_{s_x \in Q_{x,t}} \sum_{s_y \in Q_{y,t}} (1 - r) P(S_{x,t} = s_x, S_{y,t} = s_y \,|\, IBD_{1,t} = 0), \text{ where} \end{aligned}$$

$P(S_{x,t} = s_x, S_{y,t} = s_y \,|\, IBD_{1,t} = 1)$ is as in (S.1) and
$P(S_{x,t} = s_x, S_{y,t} = s_y \,|\, IBD_{1,t} = 0) = g\big(s_x; n_x, (\pi_t)\big)\, g\big(s_y; n_y, (\pi_t)\big)$. Let

$P_1 \equiv \displaystyle\sum_{s_x \in Q_{x,t}} \sum_{s_y \in Q_{y,t}} P(S_{x,t} = s_x, S_{y,t} = s_y \,|\, IBD_{1,t} = 1)$ and

$P_2 \equiv \displaystyle\sum_{s_x \in Q_{x,t}} \sum_{s_y \in Q_{y,t}} P(S_{x,t} = s_x, S_{y,t} = s_y \,|\, IBD_{1,t} = 0)$.

Then the log-likelihood for locus t can be written as

$$\ell(r; u_{x,t}, u_{y,t}, n_x, n_y, \boldsymbol{\pi}) = \log\big(r\, P_1 + (1-r)\, P_2\big) = \log(P_2) + \log\left[1 + r\left(\frac{P_1}{P_2} - 1\right)\right] \tag{S.2}$$

Since logarithmic function is monotonic, the log-likelihood for a single locus is a monotonic function of r and a maximum likelihood estimate is either 0 or 1. Thus we can explore when the function is increasing or decreasing and what is the shape of the support curve (note that the function is concave as the second derivative is negative or zero). First, consider the case when P_1/P_2 is close to 1. Then $(P_1/P_2 - 1)$ is small and $\log\left[1 + r\,(P_1/P_2 - 1)\right] \approx r\,(P_1/P_2 - 1)$ for any r, which means that the log-likelihood is approximately linear.

To compare P_1 and P_2, we can look at their components - allele frequencies and multinomial coefficients. A simple case with a single shared allele can illustrate their comparison but the concept readily extends to multiple shared alleles. P_2 has an extra factor of π_i, where a_i is a shared allele; on the other hand, it has higher factorials - depending on a combination of these factors, P_1 is either less than P_2 (log-likelihood is decreasing) - or greater (log-likelihood increasing). Smaller π_i contributes to a greater P_1/P_2 ratio (an argument for relatedness), as does a greater number of shared alleles ($|u_{xy,t}|$). This can also elucidate the "non-linear" cases where P_1/P_2 is not close to 1: if the frequencies of shared alleles are very small, the ratio is high (strong evidence of relatedness), and if there are no shared alleles, $P_1 = 0$ and log-likelihood goes to $-\infty$ at $r = 1$. This stronger evidence in support or against independence in a locus increases contribution of that locus to the overall likelihood thus having a greater effect on the relatedness estimate. Furthermore, its effect on likelihood-ratio-based inference can be even greater; for example, a shared allele with a very small population frequency in a single locus can lead to rejecting H_0: $r = 0$ even when the estimate itself is low. For practical implications, this could underline the importance of allele frequencies estimation: e.g. estimates suffering from biases in data selection, such as data not being representative of population in terms of allele frequencies, can significantly affect downstream results.

S.4 Limitations of bootstrap-based asymmetric confidence intervals

This section contains notes on constructing confidence intervals using bootstrap and applications of this method to Dcifer although it is not implemented in Dcifer directly for the reasons described below. Let $\hat{\theta}$ be an estimate of a parameter θ, for which we want to find a $1 - \alpha$ confidence interval (CI). Let CI_{lo} and CI_{up} be the values at the endpoints of such CI:

$$P\big(CI_{lo} \leqslant \theta \leqslant CI_{up}\big) = 1 - \alpha.$$

Fact:

$$P\big(q_{\alpha/2} \leqslant \hat{\theta} < q_{1-\alpha/2}\big) = 1 - \alpha,$$

where $q_{\alpha/2}$ and $q_{1-\alpha/2}$ are $\alpha/2$'th and $(1-\alpha/2)$'th quantiles of the sampling distribution. Then

$$P\big(q_{\alpha/2} - \theta \leqslant \hat{\theta} - \theta < q_{1-\alpha/2} - \theta\big) = 1 - \alpha$$
$$P\big(\hat{\theta} - (q_{1-\alpha/2} - \theta) < \theta \leqslant \hat{\theta} - (q_{\alpha/2} - \theta)\big) = 1 - \alpha$$

and

$$CI_{lo} = \hat{\theta} - (q_{1-\alpha/2} - \theta)$$
$$CI_{up} = \hat{\theta} - (q_{\alpha/2} - \theta),$$

where $q_{\alpha/2}$, $q_{1-\alpha/2}$, and θ are unknown.

Suppose we use bootstrap to approximate $q_{\alpha/2} - \theta$ with $q^*_{\alpha/2} - \hat{\theta}$ and $q_{1-\alpha/2} - \theta$ with $q^*_{1-\alpha/2} - \hat{\theta}$, where $q^*_{\alpha/2}$ and $q^*_{1-\alpha/2}$ are $\alpha/2$'th and $(1-\alpha/2)$'th quantiles of the bootstrap distribution. Then

$$CI_{lo} \approx 2\hat{\theta} - q^*_{1-\alpha/2}$$
$$CI_{up} \approx 2\hat{\theta} - q^*_{\alpha/2}.$$

Inverting the quantiles for the CI makes intuitive sense: if a sampling distribution is skewed to the right, and $\hat{\theta}$ tends to overestimate θ: $\theta - q_{\alpha/2} < q_{1-\alpha/2} - \theta$, then CI_{lo} should be further from $\hat{\theta}$ than CI_{up}. Validity of such inference rests on the bootstrap principal assumption that the distribution of $\hat{\theta}^* - \hat{\theta}$ is similar enough to the distribution of $\hat{\theta} - \theta$. However, in our case with bounded support $(0 \leqslant \theta \leqslant 1)$, these distributions can be quite different. That principal assumption would hold in the midrange, provided that the sample size is large enough, when bootstrap-based CI's are reasonably symmetric; in that case, however, they would be very similar to Wald and likelihood-ratio-based intervals.

S.5 Population allele frequencies and observed allele counts

Let N be the number of samples (infections) with $n_1, \dots, n_N$ parasite strains in them (COI). For a given locus t and a given allele $a_{t,k}$ with population frequency $\pi_{t,k}$, let $b = (b_1, \dots, b_N)$, $b_i \in \{0, 1\}$ be a binary sequence of indicators of whether that allele is present in each sample. Then, for a sample i,

$$P(b_i = 1) = 1 - (1 - \pi_{t,k})^{n_i}, \text{ and}$$

$$L(\pi_{t,k}; b) = \prod_{i=1}^{N} \Big[b_i \big(1 - (1 - \pi_{t,k})^{n_i}\big) + (1 - b_i)(1 - \pi_{t,k})^{n_i} \Big] = \prod_{\{i: b_i = 1\}} (1 - q^{n_i}) \prod_{\{j: b_j = 0\}} q^{n_j}$$
$$\ell(\pi_{t,k}; b) = \log \big[L(\pi_{t,k}; b) \big] = \sum_{\{i: b_i = 1\}} \log (1 - q^{n_i}) + \log q \sum_{\{j: b_j = 0\}} n_j,$$

where $q = 1 - \pi_{t,k}$.

If $n_1 = \ldots = n_N = 1$ (all the infections are monoclonal), $E\big[\sum_{i=1}^N b_i\big] = N\pi_{t,k}$; otherwise $E\big[\sum_{i=1}^N b_i\big] > N\pi_{t,k}$. Naive estimation of allele frequencies as normalized proportions of number of samples with each allele at a given locus will result in underestimating frequencies of common alleles and overestimating rare allele frequencies. In turn, that would lead to higher heterozygosity, and, consequently, to overestimating relatedness between infections. The reason for such overestimation is that sharing of common alleles would be considered less likely to have occured by chance (as opposed to descent) than it actually is. Therefore it is advisable to adjust allele frequencies estimation to account for complex infections.

S.6 Supplementary figures

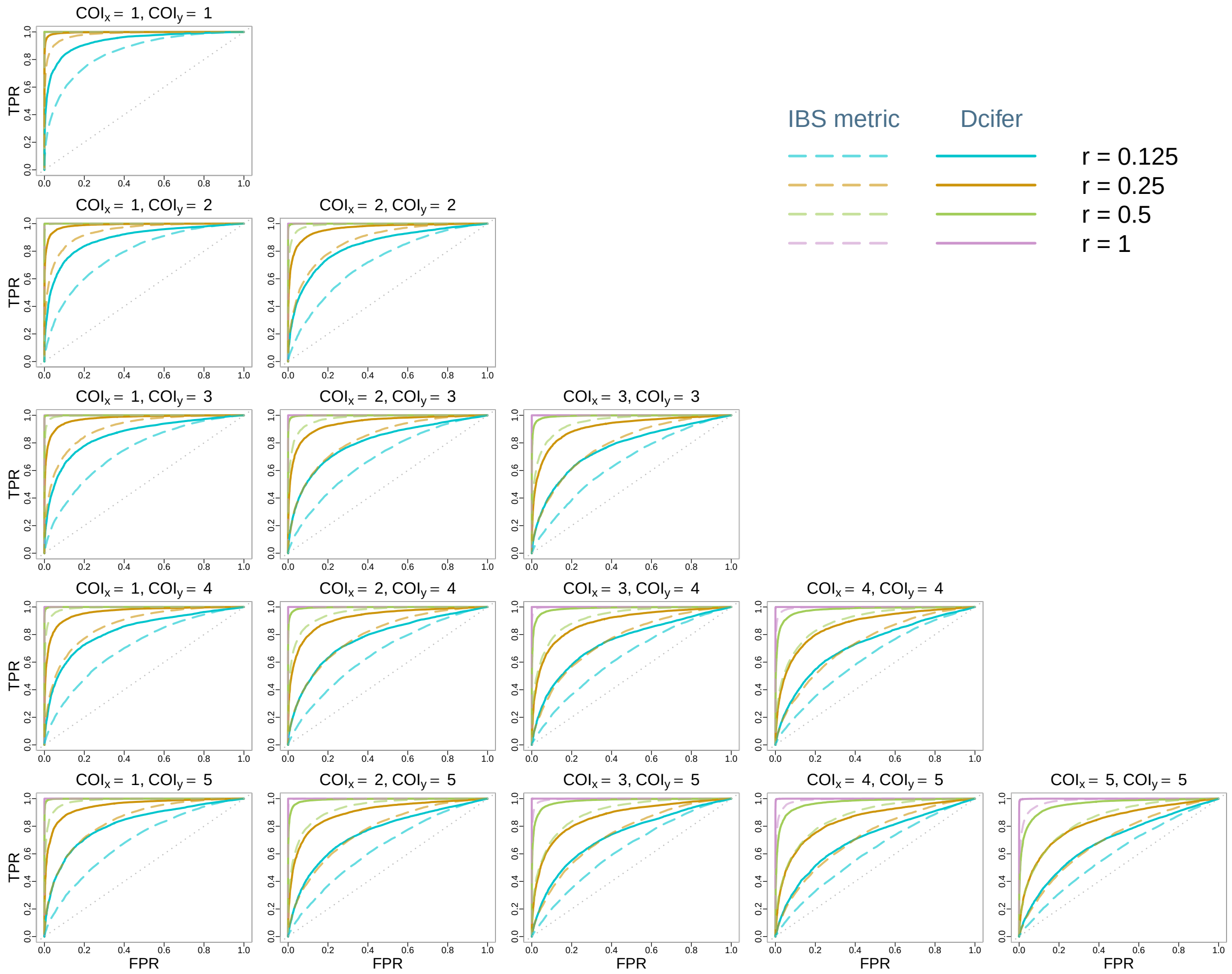

Figure S.1: Receiver operating characteristic curves for Dcifer relatedness estimator $\hat{r}$ and IBS similarity metric, classification accuracy for related when compared to $r = 0$. Data were simulated using a panel of 91 microhaplotypes; simulations were performed for five values of r and for COI combinations ranging between 1 and 5; true values of COI and population allele frequencies were used for Dcifer.

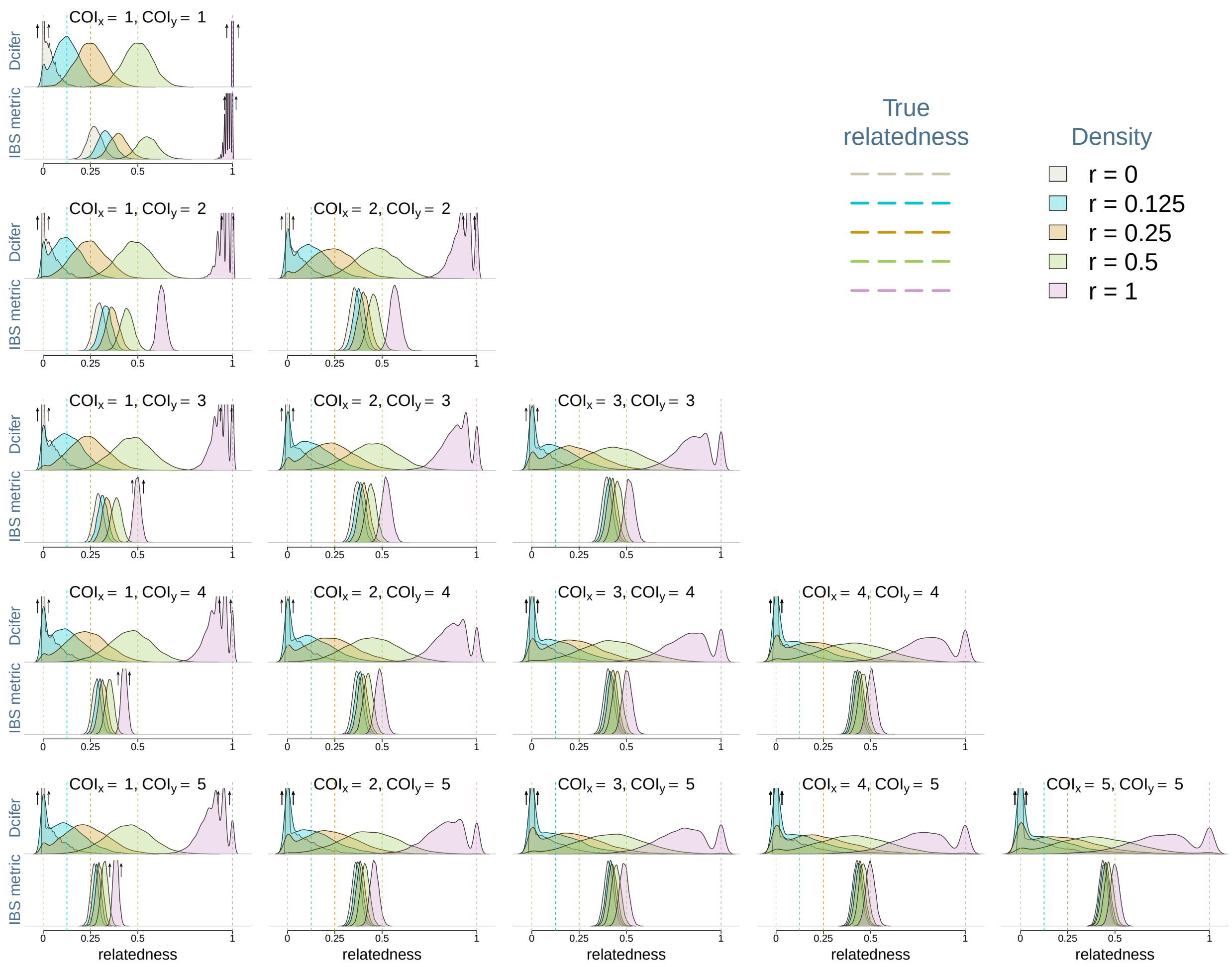

Figure S.2: Densities of Dcifer relatedness estimator $\hat{r}$ and IBS similarity metric results obtained from data simulated with genotyping errors ($\epsilon = 0.05$, $\lambda = 0.01$) using a panel of 91 microhaplotypes. Simulations were performed for five values of r and for COI combinations ranging between 1 and 5. Estimated COI and population allele frequencies were used for Dcifer.

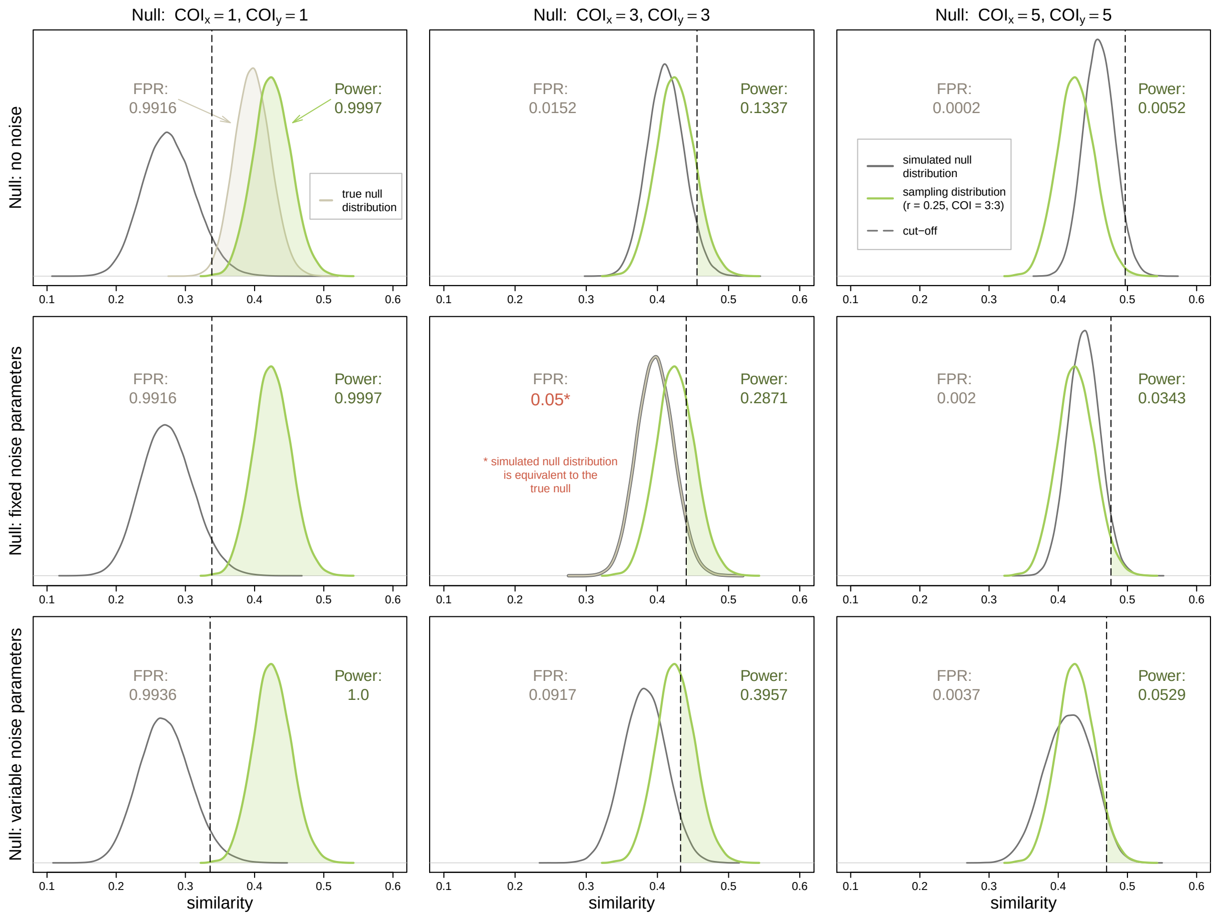

Figure S.3: Hypothesis testing with different reference (simulated null) distributions for the IBS metric applied to related infections ($r = 0.25$) with COI of 3. Vertical dotted line in each panel represents a rejection cut-off determined by the 0.95-quantile of the corresponding null (the area under the density curve in dark grey to the left of the line is equal to 0.95). The green curve of the sampling distribution and the light beige curve of the true null displayed in the top left panel are the same for each panel. Note the narrow range of the values (x-axis range represents only part of the $[0, 1]$ support). Depending on the assumed null distribution, the power ranges between 0.0052 and 1 and the false positive rate (FPR) - between 0.0002 and 0.9936, highlighting how sensitive this inferential approach is to assumptions about COI and error, in contrast to the likelihood ratio approach available using Dcifer.

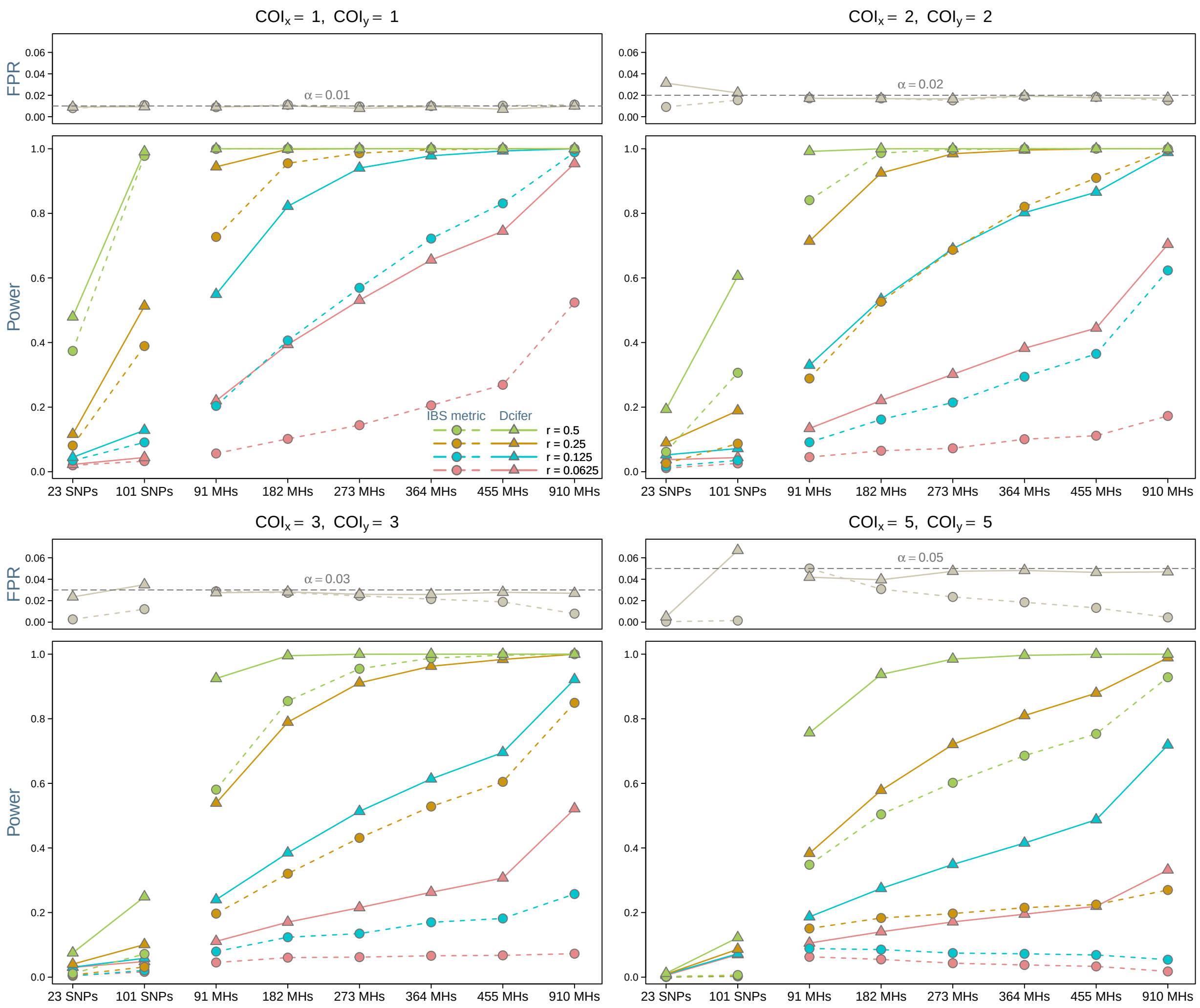

Figure S.4: Detecting related infections. False positive rate and statistical power of a test $H_0: r = 0$ at significance level $\alpha = 0.01 \times min(COI_x, COI_y)$ are shown. Simulations were performed with genotyping error, and COI were estimated from these data. Single nucleotide polymorphism (SNP) and microhaplotype (MH) panels were used as a basis for simulations.

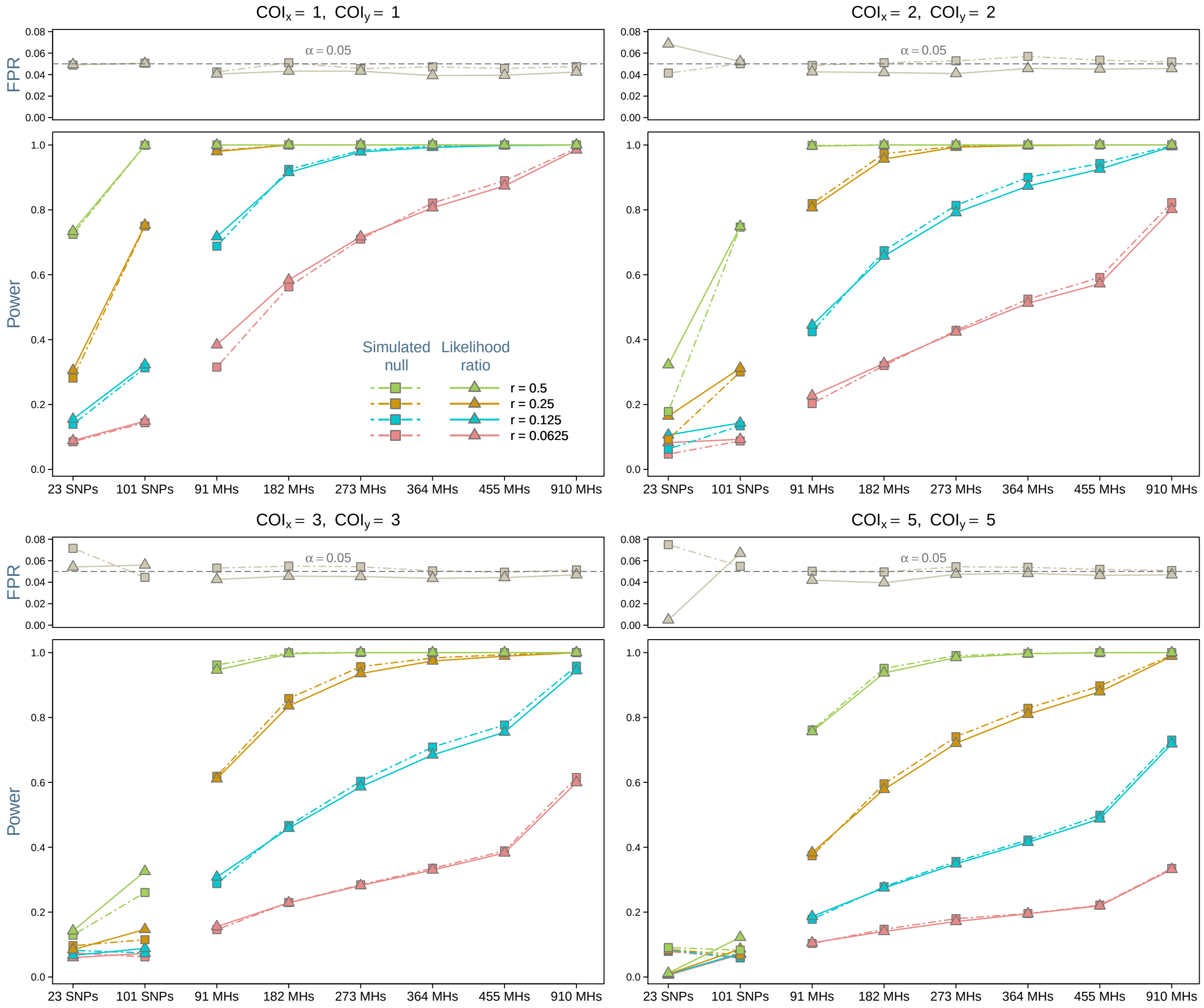

Figure S.5: Comparison of two inferential approaches within Dcifer: simulated null distributions vs likelihood ratio. False positive rate and statistical power of a test H_0: $r = 0$ at significance level $\alpha = 0.05$ are shown. Simulations were performed with genotyping error, and COI were estimated from these data.

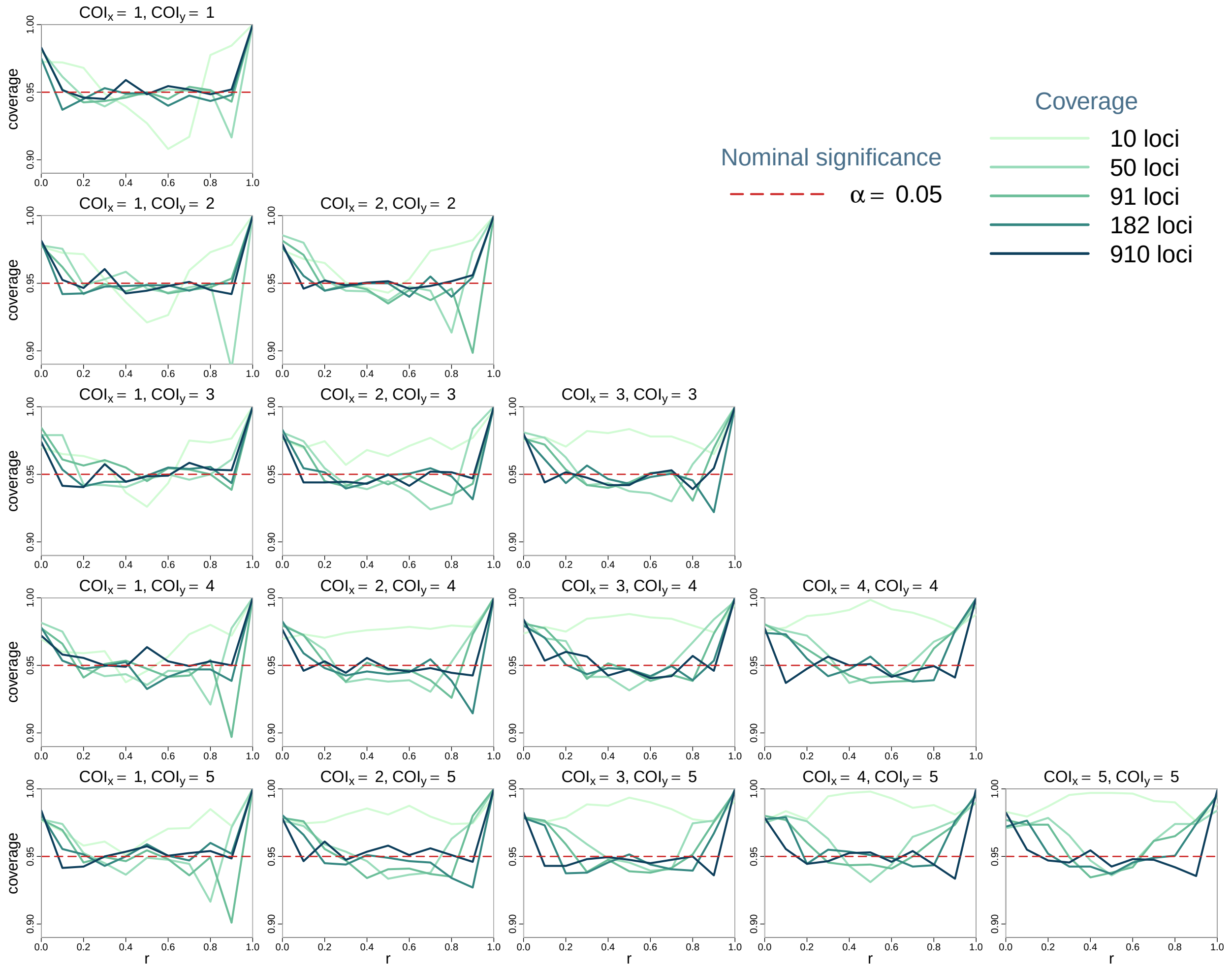

Figure S.6: Coverage for likelihood-ratio-based 95% confidence intervals produced by Dcifer.

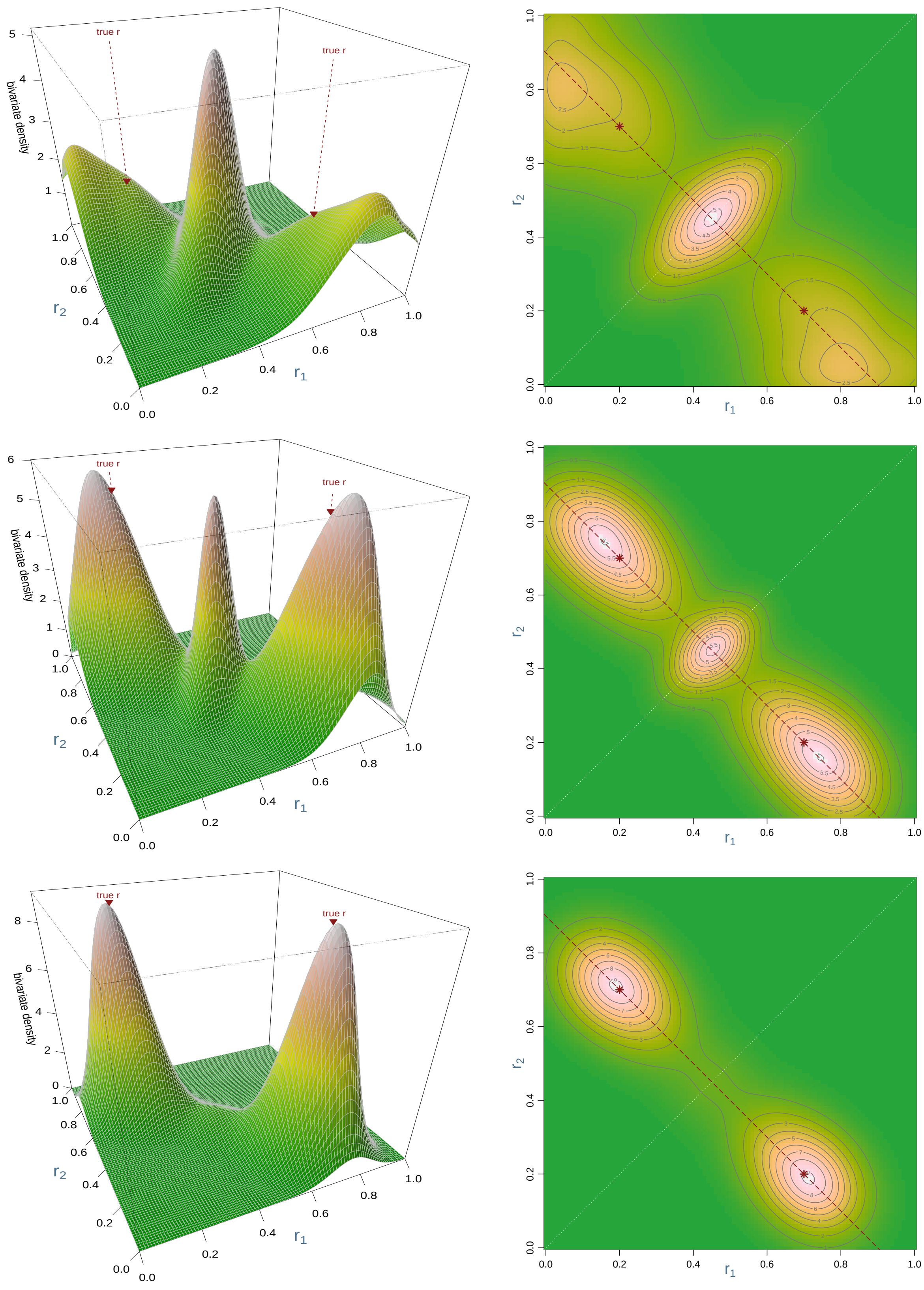

Figure S.7: Sampling distribution for $M = 2$, $r_1 = 0.2, r_2 = 0.7$, $n_x = n_y = 3$. True values are indicated by red stars on the contour plots; red dotted line represents $r_{total} = r_1 + r_2$. Top row: 91 loci, middle row: 273 loci, bottom row: 910 loci. We can see two tendencies in the estimation of multiple parameters: one is for the estimates to be equal and another - to separate them further, pulling some estimates toward 0 or 1.

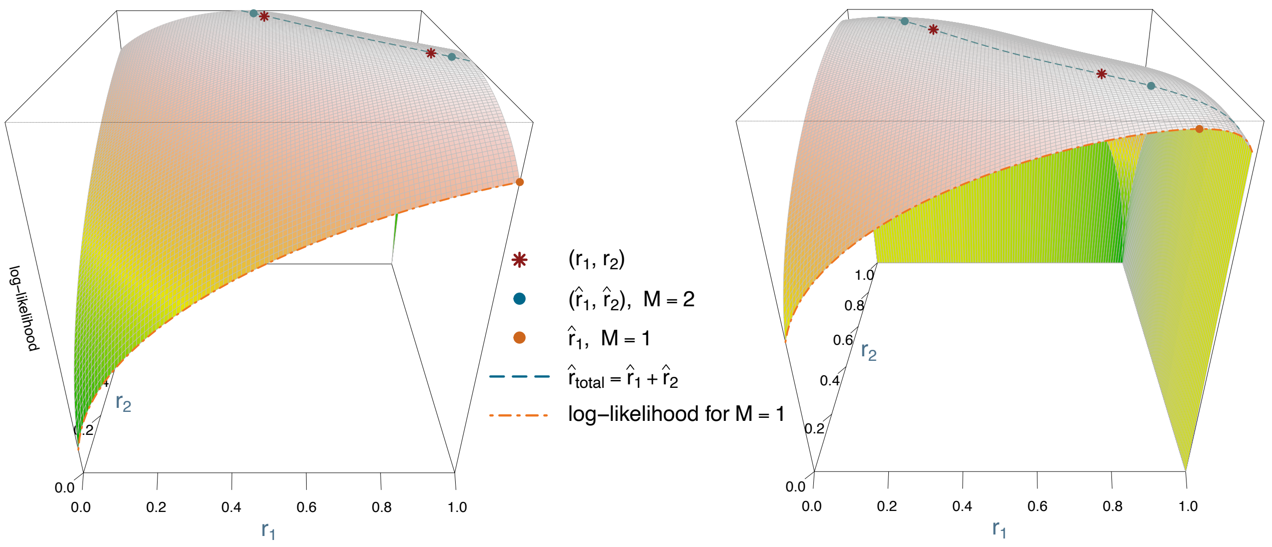

Figure S.8: Examples of log-likelihood surfaces for two pairs of related infections with COI of 3, $M = 2$. The true parameter values (r_1, r_2) are $(0.48, 0.92)$ in the left panel and $(0.31, 0.74)$ in the right.

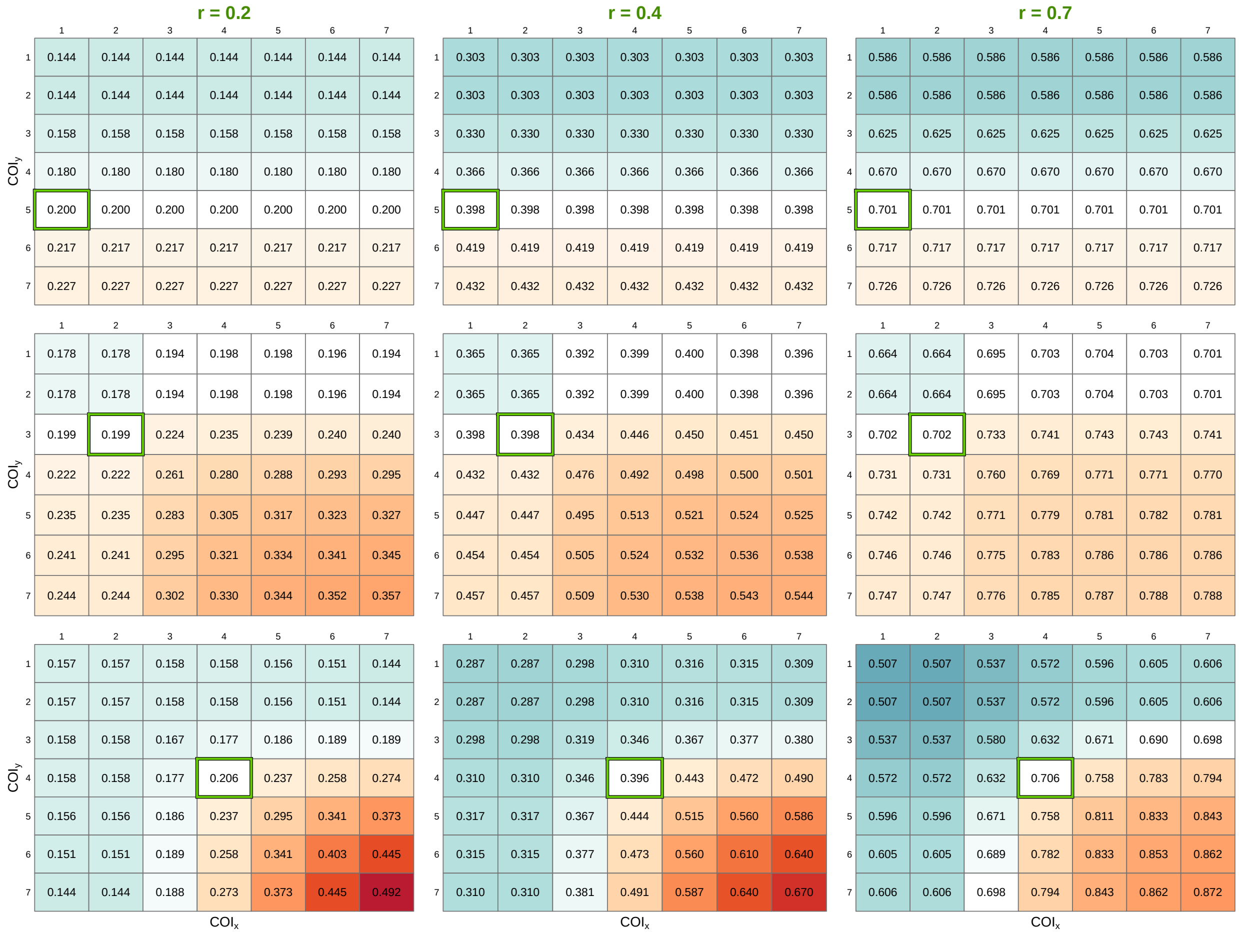

Figure S.9: Effect of COI misspecifications on relatedness estimates. Each panel represents a matrix whose entries are average estimates obtained with a corresponding COI combination. True COI is outlined in green; background color of each entry corresponds to the deviation from the true r value.

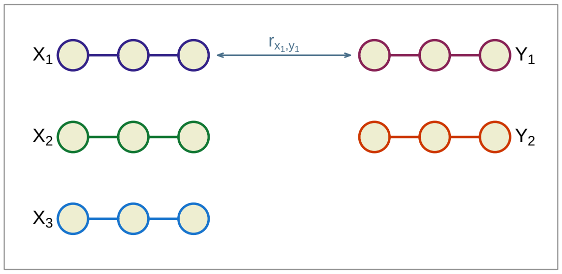

(a) No intrahost relatedness.

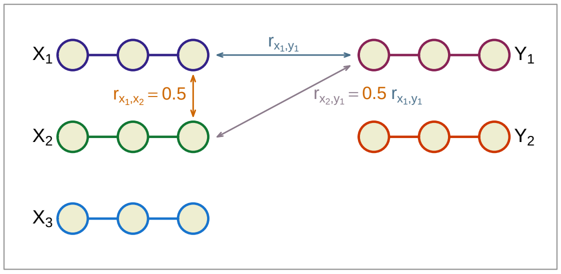

(b) Strains X_1 and X_2 are related.

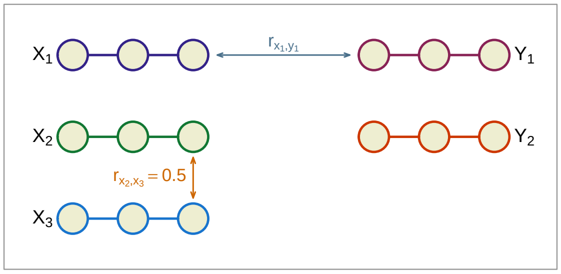

(c) Strains X_2 and X_3 are related.

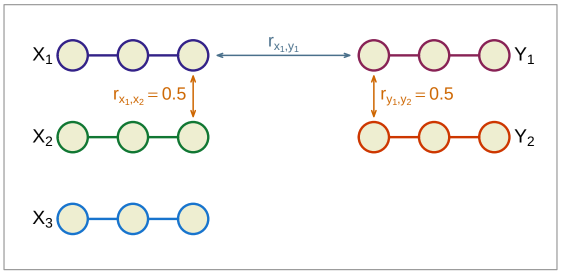

(d) Strains X_1 and X_2 are related, strains Y_1 and Y_2 are related.

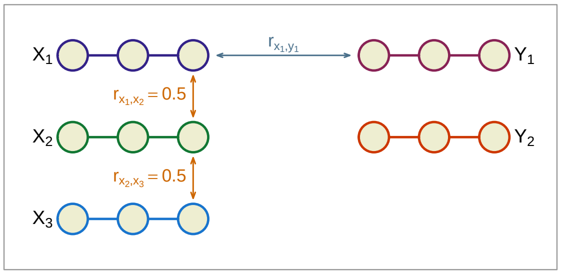

(e) Strains X_1 and X_2 are related, strains X_2 and X_3 are related.

Figure S.10: Diagram of intrahost relatedness simulations using an example with COI of 3 and 2. Five scenarios are compared, each with the same level of relatedness between strains represented by X_1 and Y_1, which is not changed by the induced intrahost relatedness in (b-e). Horizontal chains of connected circles represent haplotypes.

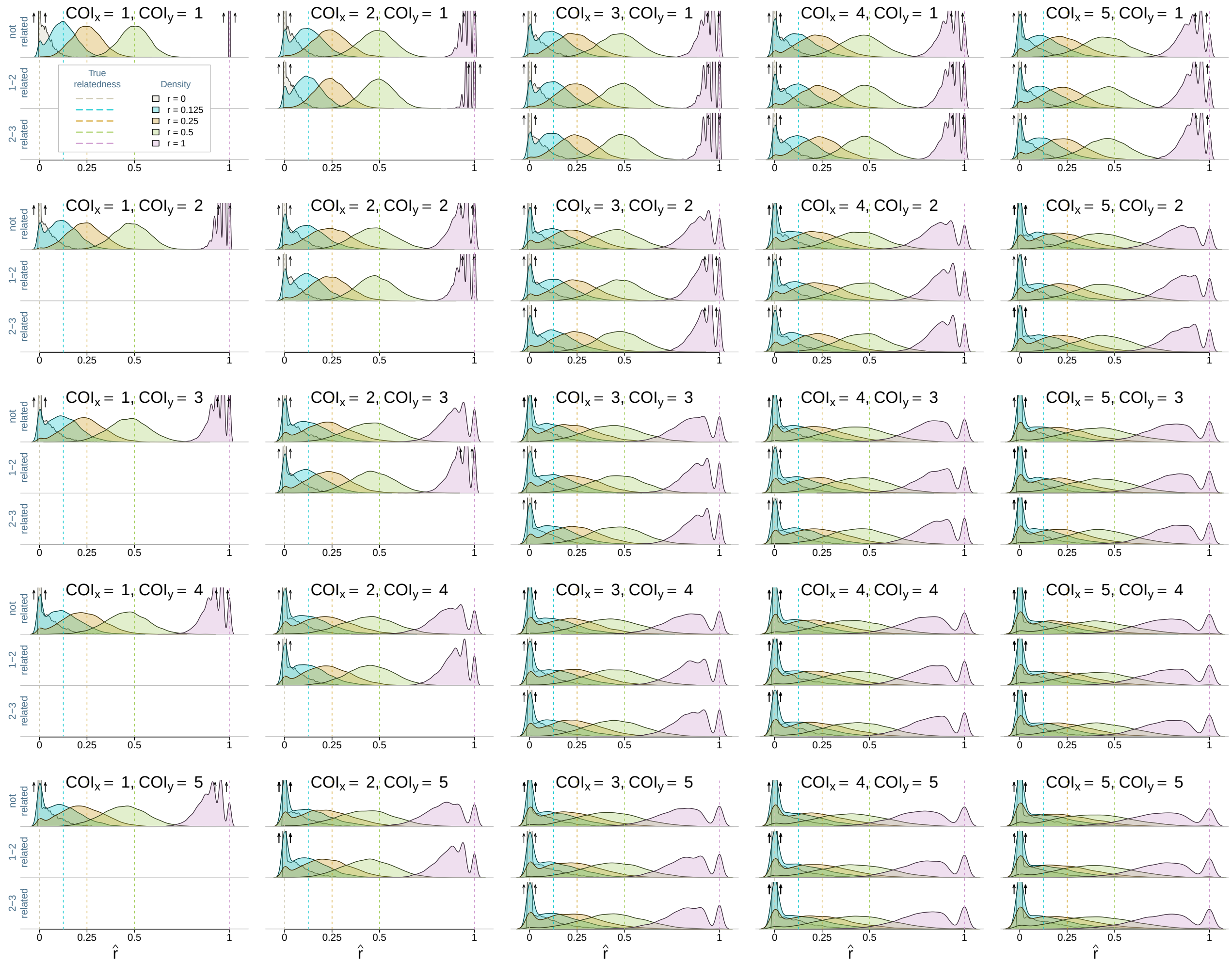

Figure S.11: Comparison of sampling distributions across three intrahost relatedness scenarios: 1) no intrahost relatedness, 2) strains X_1 and X_2 are related, 3) strains X_2 and X_3 are related (as illustrated in Figures S.10a, S.10b, and S.10c).

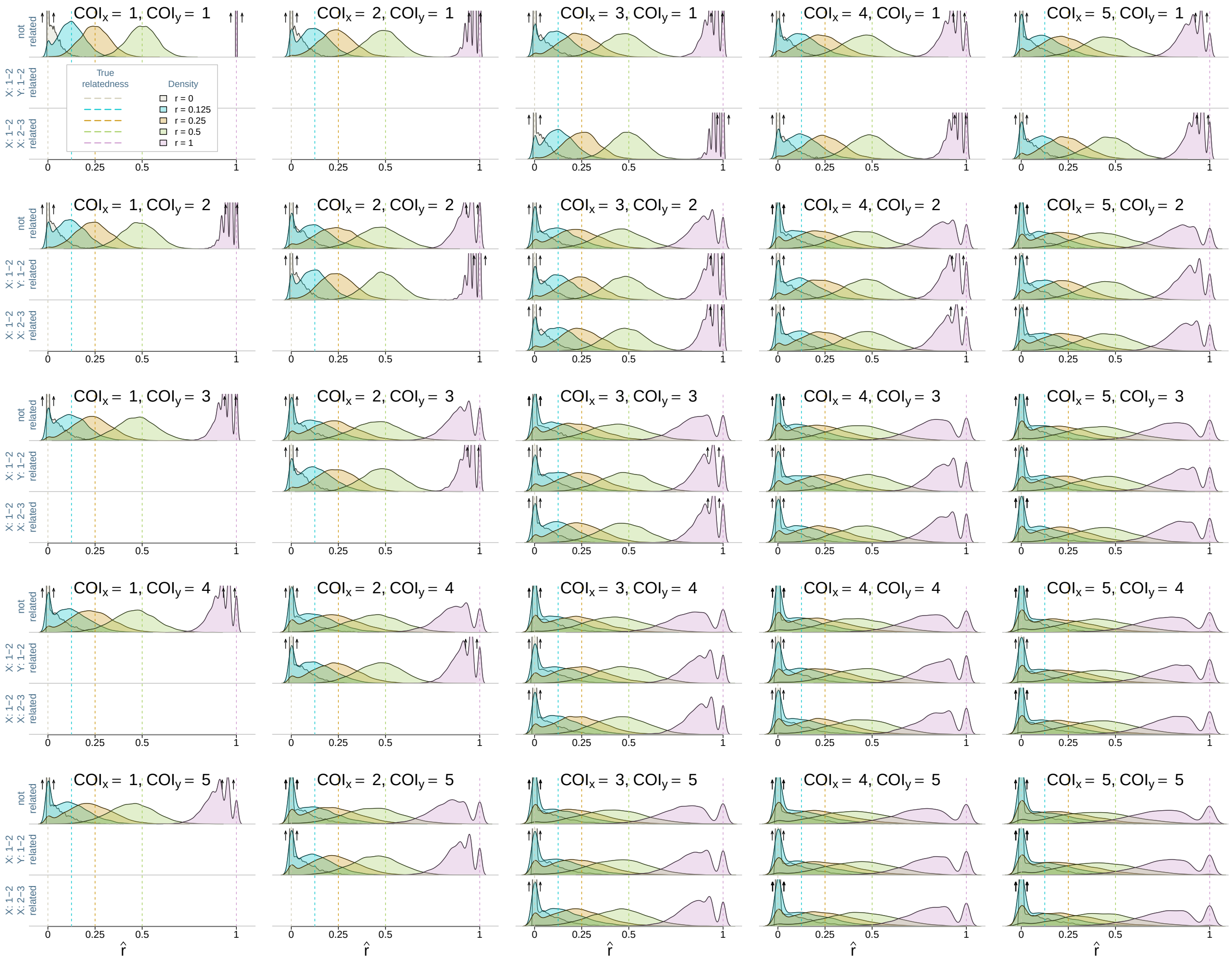

Figure S.12: Comparison of sampling distributions across three intrahost relatedness scenarios: 1) no intrahost relatedness, 2) strains X_1-X_2 and Y_1-Y_2 are related, 3) strains X_1, X_2 and X_3 are related, with X_1-X_3 relatedness being a result of induced X_1-X_2 and X_2-X_3 relatedness (as illustrated in Figures S.10a, S.10d, and S.10e).
