# Supplementary material for: Dcifer: an IBD-based method to calculate genetic distance between polyclonal infections
Source: Genetics. 2022 Aug 24;222(2):iyac126. doi: 10.1093/genetics/iyac126 (PMC9526043; doi:10.1093/genetics/iyac126)
Supplement: iyac126_Supplemental_Material_File_2 [file iyac126_supplemental_material_file_2.pdf]

Generating multisets with mixed radix incrementing algorithm

https://github.com/innager/mirsa

1 Concept

Many problems that require traversing all combinations of a given type that satisfy a number of conditions and performing computations/manipulations on each combination can be conveniently framed in terms of **multisets**. A multiset M, which is a collection of elements that do not need to be distinct (unlike elements in a set), may be defined as a 2-tuple (A, m), where A is some set of elements that includes those present in M and $m : A \to \mathbb{Z}^{\geqslant}$ is a function from A to the set of non-negative integers giving multiplicity of each element in A. Thus A is a superset of the support of M, where $Supp(M)$ is a set of unique elements of M; $A \supseteq Supp(M)$.

If a combination is represented by a multiset, all combinations that need to be enumerated can form a collection of multisets. Let A be a finite set containing all the possible elements of the multisets in this collection; in addition, assign some arbitrary order to the elements of A. Then each multiset can be represented by a sequence of multiplicities of the ordered elements of A, i.e. the number of occurences of each element in a corresponding multiset. For a collection of multisets, these sequences are of the same length and their elements are finite non-negative integers. In turn, each sequence can be thought of as a representation of a **number** in some positional numeral system, with each element of the sequence being a single digit of that number (regardless of how many digits are used for that element in, for example, the decimal numeral system). Then all the sequences in the collection can be translated into distinct numbers in the same numeral system.

To account for all the combinations, it might be useful to impose some kind of ordering on them and, consequently, a rule prescribing how to move from one combination to the next. Ultimately, finding a rule that would allow one to efficiently traverse all the necessary combinations is the problem to solve. Since a combination can be represented by a single number, moving from that combination to the next can be as simple as incrementing or decrementing that number or, if there are constraints (e.g. a bound on a sum of the digits of the number), moving to the closest "allowed" number. Because different representations of a combination described above are bijective, the "next" number uniquely defines a combination in the collection. Figure 1 provides a diagram of how the chain of these representations can be used to move from a given combination to the next one.

Positional numeral systems

A base, or **radix**, is the number of unique digits at each position: the smallest digit is 0 and the greatest is radix minus 1.

- Standard systems: fixed base – same base at each position (e.g. decimal system: base 10, binary system: base 2);

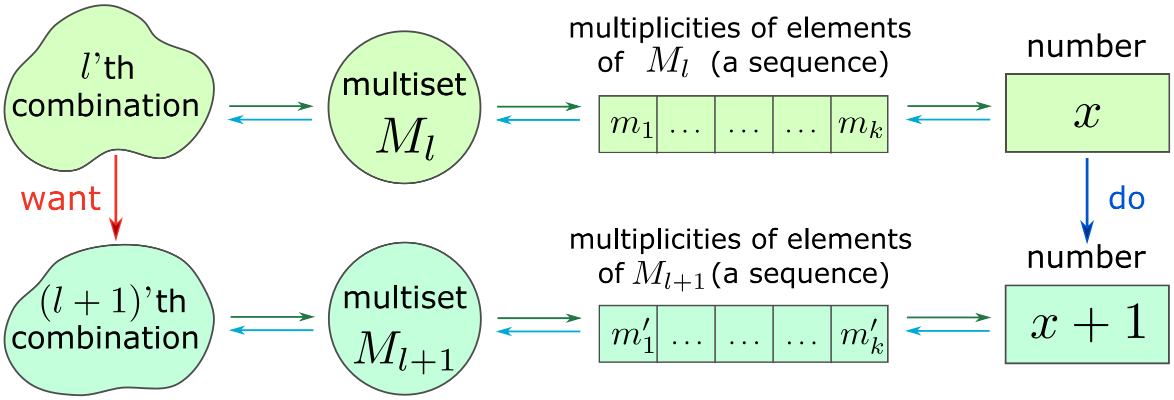

Figure 1: Progressing through combinations of some type by converting a combination to a number in some positional numeral system through a chain of different representations, incrementing that number, and converting it back to a combination of the same type.

- Non-standard systems: varying base – different bases are allowed at each position. Example – time measurement:

units	weeks	days	hours	minutes	seconds
radix	∞	7	24	60	60

or

units	weeks	days	am/pm	hours	minutes	seconds
radix	∞	7	2	12	60	60

The number represented by a sequence of digits $(a_n, a_{n-1}, \ldots, a_1, a_0)$ (usually written as $a_n a_{n-1} \cdots a_1 a_0$) in a numeral system with radices $(b_n, b_{n-1}, \ldots, b_1, b_0)$, $0 < b_i < \infty \ \ \forall\, i$ is equal to $a_0 + \sum_{i=1}^{n} a_i \prod_{j=1}^{i} b_j$.

Incrementing

Incrementing a number in a **mixed radix** system can follow the same principal as in standard systems: going from right to left, find the first position that can be incremented, add 1, and set all the positions to the right of that position to 0 (Figure 2). The star in the top panel of Figure 2 (representing x) indicates the position to be incremented. Note that having positions with base 1 can be useful in some situations. The digital representations of x and $x+1$ in the same mixed radix numeral system are shown in Figure 3 (in decimal system, their values are $7,917,695$ and $7,917,696$).

Constraints

The incrementing procedure described above is outlined in Knuth [2011] Algorithm M (Mixed-radix generation) for generating all n-tuples. By incorporating some constraints into that algorithm and adding modifications, we can efficiently solve a surpising number of problems. A very useful constraint would be an upper limit on cardinality of the multisets we want to generate; we might also want to set a lower non-zero bound on a digit at each position. A modification that includes

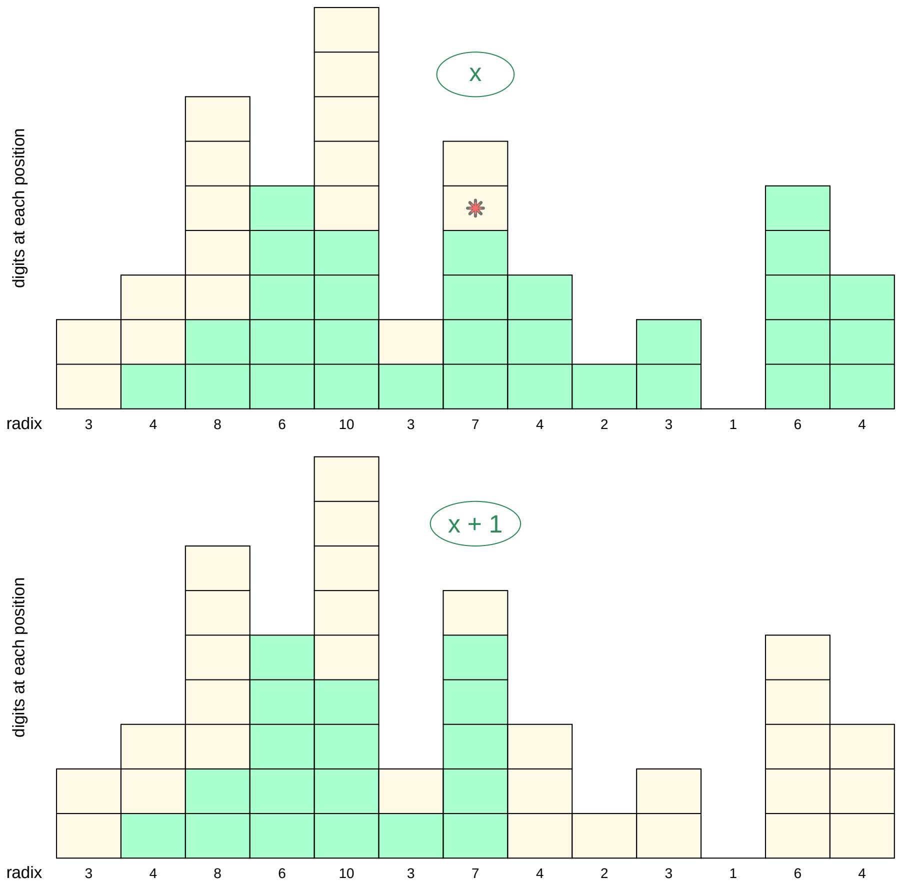

Figure 2: Incrementing in a mixed radix system: graphical representation.

x	0	1	2	5	4	1	4	3	1	2	0	5	3
radix	3	4	8	6	10	3	7	4	2	3	1	6	4
x + 1	0	1	2	5	4	1	5	0	0	0	0	0	0

Figure 3: Incrementing in a mixed radix system: digital representation.

position-associated weights could accomodate another set of problems. Here we extend this simple and intuitive algorithm to include the cardinality constraint, look at other possible extensions, and illustrate the utility of this approach with the following examples:

- Generate all the collections of non-unique elements of a given size n from a set A of their unique elements (all the multisets M_l such that $|M_l| = n$ and $Supp(M_l) = A$);
- Find all the sub-collections of a given collection B with a cardinality constraint n (all the multisets M_l such that $M_l \subseteq B$ and $|M_l| \leqslant n$);
- Find all the binary sequences with elements in $\{0, 1\}$ of length k where the number of 1's does not exceed $n \leqslant k$;
- Enumerate all the collections of elements whose sum is equal to a given number n.

2 Algorithm

2.1 Main constraint

Let $\boldsymbol{b} = (b_1, \ldots, b_k)$ be radices, $\boldsymbol{m} = (m_1, \ldots, m_k)$ be the digits (values) at each position, S_{max} – the maximum sum, $\sum_{i=1}^{k} m_i \leqslant S_{max}$, and $\boldsymbol{d}_{max} = (d_{max,1}, \ldots, d_{max,k})$ – the largest possible digits (maximum values) at each position, $d_{max,i} = b_i - 1$. Let $\boldsymbol{m}_{lim}$ be the last sequence to be generated, representing the greatest number satisfying the maximum sum condition (e.g. for $\boldsymbol{d}_{max} = (3, 5, 4, 7)$ and $S_{max} = 9$, $\boldsymbol{m}_{lim} = c(3, 5, 1, 0)$).

Algorithm 1: Mixed radix incrementing, main version

```
Input  : An integer S_max,
         an integer k,
         a sequence d_max = (d_max,1, ..., d_max,k) of integers,
         a function f_lim(S, k, d);                       // to calculate m_lim
Output: A list C of sequences satisfying S_max condition

m_lim ← f_lim(S_max, k, d_max);
S ← 0;
m ← k-tuple (0, ..., 0);
C ← {m};                                                  // list of length 1
i ← k;
while m ≠ m_lim do
    if m_i = d_max,i ∨ S = S_max then
        S ← S − m_i;
        m_i ← 0;
        i ← i − 1;
    else
        m_i ← m_i + 1;
        S ← S + 1;
        i ← k;
        append {m} to C;
    end
end
return C
```

Including a lower bound S_{min} on the sum as a constraint would be very similar to incorporating an upper bound in the algorithm above. Instead of a sequence of zeros, the initial sequence $\boldsymbol{m}_{init}$ would represent the smallest number satisfying S_{min} condition, e.g. for $\boldsymbol{d}_{max} = (3, 9, 1, 2)$ and $S_{min} = 5$, $\boldsymbol{m}_{init} = (0, 2, 1, 2)$.

2.2 Versions and modifications

In addition to specifying $d_{max,i}$ for each position, it might be useful for some problems to specify $d_{min,i}$ instead of using 0 as the smallest value. The simplest example would be to generate multisets with the same support, where $m(a_i) > 0 \;\; \forall i$; in other examples varying $d_{min,i}$ from position to position might be needed. The straightforward solution in that case would be to run the algorithm with a new sequence $\boldsymbol{d}'_{max}$, where $d'_{max,i} = d_{max,i} - d_{min,i} \;\; \forall\, i$, and $S'_{max} = S_{max} - \sum_{i=1}^{k} d_{min,i}$, then add $d_{min,i}$ to m_i for each i in all resulting multisets. Alternatively, it might be a little more

efficient to rewrite the algorithm substituting $d_{min,i}$ for 0's and calculating the "largest number" taking $\boldsymbol{d}_{min}$ into account.

Another possible version could have a constraint W_{max}, such that $\sum_{i=1}^{n} a_i \leqslant W_{max}$ instead of S_{max} (a sum of all elements in a multiset instead of its cardinality). In this case, each position could be assigned a weight w_i (e.g. $w_i = W_{max} - i + 1$) and the condition rewritten as $\sum_{i=1}^{l} m_i w_i \leqslant W_{max}$.

Algorithm 2: Mixed radix incrementing, weighted sum version

Input : An integer W_{max}.
Output: A collection C of sequences satisfying W_{max} condition.

```
k ← W_max;
S ← 0;
m ← k-tuple (0, ..., 0);
C ← {m};
i ← k;
while m_1 = 0 do
    w ← W_max − i + 1;                          // weight of the position
    if S + w > W_max then
        S ← S − w m_i;
        m_i ← 0;
        i ← i − 1;
    else
        m_i ← m_i + 1;
        S ← S + w;
        i ← k;
        append {m} to C;
    end
end
return C
```

Using the mixed radix incrementing approach for this particular problem is not necessarily the most efficient way to traverse the combinations, but it has an advantage of being intuitive and easily constructed without any additional knowledge.

3 Example Problems

3.1 Multisets of given cardinality and support

Generate all multisets M_l of cardinality n with $Supp(M_l) = A$, where A is a given set: $|M_l| = n \ \forall l$; if $x \in A$, then $x \in M_l$; if $|A| > n$, $\mathbb{M} = \varnothing$ (see Table 1 for an example). A problem like this can arise, for example, when a multiset of a known cardinality that we are interested in is unobserved and only the set of its unique elements is observed.

set A	multiset M_l	multiplicities of M_l
$\{a_1, a_2, a_3\}$	$\{a_1, a_1, a_1, a_2, a_3\}$	3 1 1
	$\{a_1, a_1, a_2, a_2, a_3\}$	2 2 1
	$\{a_1, a_1, a_2, a_3, a_3\}$	2 1 2
	$\{a_1, a_2, a_2, a_2, a_3\}$	1 3 1
	$\{a_1, a_2, a_2, a_3, a_3\}$	1 2 2
	$\{a_1, a_2, a_3, a_3, a_3\}$	1 1 3

Table 1: Multisets of support A, $|A| = 3$, and cardinality 5.

The number of multisets satisfying these conditions is called a multiset coefficient (or multiset number); it is a function of n and $k \equiv |A|$ and is equal to $\binom{n-1}{k-1}$. It can also be defined recursively:

$$f(0, y) = 1 \;\; \forall\, y \in \mathbb{Z}^{\geqslant}$$
$$f(x, 0) = 0 \;\; \forall\, x \in \mathbb{Z}^{>}$$
$$f(x, y) = f(x-1, y) + f(x, y-1)$$

Then the multiset coefficient is equal to $f(k-1, n-k+1)$.

```
> mcoef <- function(x, y) {
+   if (x == 0) return(1)
+   res <- 0
+   for (yi in y:1) {                      # y:1 for clarity
+     res <- res + mcoef(x - 1, yi)
+   }
+   return(res)
+ }
> n <- 12
> k <- 6
> choose(n - 1, k - 1)

[1] 462

> mcoef(k - 1, n - k + 1)

[1] 462
```

The algorithm outputs multisets of cardinality less than or equal to a given number, so to get the multisets of the given cardinality only, we run the algorithm with one position removed ($k' = k - 1$) and set $d_{min,i} = 1 \;\forall\, i$. Note that for this problem, the base is the same for all the positions and is equal to $n - k + 1$. The output is a matrix, in which each multiset M_l is represented by a column with multiplicities of elements of A (the third column in Table 1). Below we generate multisets for $n = 8$ and $k = 4$; the last row is added after running the algorithm to bring the cardinality of each multiset to n: $\sum_{i=1}^{k} m_i = n$.

```
> n <- 8
> k <- 4
```

multiset B	$M_l \subseteq B$, $\lvert M_l\rvert \leqslant \lvert B\rvert$	$M_l \subseteq B$, $\lvert M_l\rvert \leqslant 2$	multiplicities of M_l
$\{a_1, a_1, a_2\}$	$\varnothing$	$\varnothing$	0 0
	$\{a_1\}$	$\{a_1\}$	1 0
	$\{a_2\}$,	$\{a_2\}$,	0 1
	$\{a_1, a_1\}$	$\{a_1, a_1\}$	2 0
	$\{a_1, a_2\}$	$\{a_1, a_2\}$	1 1
	$\{a_1, a_1, a_2\}$		2 1

Table 2: Multisets included in a multiset B of different conditions on cardinality.

```
> # Function mirsa() (MIxed Radix System Algorithm) runs the main version
> # of the algorithm
> vmat <- mirsa(rep(n - k, k - 1), summax = n - k) + 1
> rbind(vmat, n - colSums(vmat))

     [,1] [,2] [,3] [,4] [,5] [,6] [,7] [,8] [,9] [,10] [,11] [,12] [,13] [,14]
[1,]    1    1    1    1    1    1    1    1    1     1     1     1     1     1
[2,]    1    1    1    1    1    2    2    2    2     3     3     3     4     4
[3,]    1    2    3    4    5    1    2    3    4     1     2     3     1     2
[4,]    5    4    3    2    1    4    3    2    1     3     2     1     2     1
     [,15] [,16] [,17] [,18] [,19] [,20] [,21] [,22] [,23] [,24] [,25] [,26]
[1,]     1     2     2     2     2     2     2     2     2     2     2     3
[2,]     5     1     1     1     1     2     2     2     3     3     4     1
[3,]     1     1     2     3     4     1     2     3     1     2     1     1
[4,]     1     4     3     2     1     3     2     1     2     1     1     3
     [,27] [,28] [,29] [,30] [,31] [,32] [,33] [,34] [,35]
[1,]     3     3     3     3     3     4     4     4     5
[2,]     1     1     2     2     3     1     1     2     1
[3,]     2     3     1     2     1     1     2     1     1
[4,]     2     1     2     1     1     2     1     1     1
```

3.2 Multisets $M_l \subseteq B$, $|M_l| \leqslant n$

Given a multiset B and a number n, we want to find all multisets that are included in B: $\forall x \in A$, $m_{M_l}(x) \leqslant m_B(x)$ of cardinality $|M_l| \leqslant n$ (Table 2).

First, consider the case when $n = |B|$, which means it's a simple case of incrementing without any constrains. The number of such multisets is $\prod_{i=1}^{k} b_i$ and the output is all the multisets included in B (in Table 2 example, $b_1 = 3$ and $b_2 = 2$). If $n < |B|$, some "numbers" will be skipped, which is handled by one of the conditions in the loop; the number of multisets can be calculated by summing up the numbers for all cardinalities up to n. Below we generate multisets with $b_1 = 3$, $b_2 = 5$, and $b_3 = 2$ – first with no bound on cardinality, and then with $S_{max} = 5$:

```
> # multiplicities for B
> mB <- c(2, 4, 1)
```

sequence															
1	2	3	4	5	6	7	8	9	10	11	12	13	14	15	16
0	1	0	0	0	0	1	1	1	1	0	0	0	0	0	0
0	0	1	0	0	0	1	0	0	0	1	1	1	0	0	0
0	0	0	1	0	0	0	1	0	0	1	0	0	1	1	0
0	0	0	0	1	0	0	0	1	0	0	1	0	1	0	1
0	0	0	0	0	1	0	0	0	1	0	0	1	0	1	1

Table 3: Binary sequences of length 5 and sum no greater than 2.

```
> mirsa(mB)                 # no constraints: n = 7

     [,1] [,2] [,3] [,4] [,5] [,6] [,7] [,8] [,9] [,10] [,11] [,12] [,13] [,14]
[1,]    0    0    0    0    0    0    0    0    0     0     1     1     1     1
[2,]    0    0    1    1    2    2    3    3    4     4     0     0     1     1
[3,]    0    1    0    1    0    1    0    1    0     1     0     1     0     1
     [,15] [,16] [,17] [,18] [,19] [,20] [,21] [,22] [,23] [,24] [,25] [,26]
[1,]     1     1     1     1     1     1     2     2     2     2     2     2
[2,]     2     2     3     3     4     4     0     0     1     1     2     2
[3,]     0     1     0     1     0     1     0     1     0     1     0     1
     [,27] [,28] [,29] [,30]
[1,]     2     2     2     2
[2,]     3     3     4     4
[3,]     0     1     0     1

> mirsa(mB, summax = 5)    # cardinality constraint: n = 5

     [,1] [,2] [,3] [,4] [,5] [,6] [,7] [,8] [,9] [,10] [,11] [,12] [,13] [,14]
[1,]    0    0    0    0    0    0    0    0    0     0     1     1     1     1
[2,]    0    0    1    1    2    2    3    3    4     4     0     0     1     1
[3,]    0    1    0    1    0    1    0    1    0     1     0     1     0     1
     [,15] [,16] [,17] [,18] [,19] [,20] [,21] [,22] [,23] [,24] [,25] [,26]
[1,]     1     1     1     1     1     2     2     2     2     2     2     2
[2,]     2     2     3     3     4     0     0     1     1     2     2     3
[3,]     0     1     0     1     0     0     1     0     1     0     1     0
```

3.3 Binary sequences with a bound on the sum

Produce all binary sequences $(a_1, \ldots, a_k)$ of length k and $\sum_{i=1}^{k} a_i \leqslant n$ (Table 3). The solution of course is equivalent to generating combinations of l out of k elements, $l \leqslant n$ with the corresponding number of such sequences equal to $\binom{k}{0} + \binom{k}{1} + \cdots + \binom{k}{n}$. The mixed radix incrementing algorithm, however, might provide a more efficient solution, which in this case is as simple as incrementing in binary numeral system but with an optional constraint on the sum of the digits.

As an example, we generate binary sequences of length 5 and the sum bounded by 3:

```
> n <- 3
> k <- 5
```

```
> sum(choose(k, 0:n))  # number of multisets

[1] 26

> mirsa(rep(1, k), n)

     [,1] [,2] [,3] [,4] [,5] [,6] [,7] [,8] [,9] [,10] [,11] [,12] [,13] [,14]
[1,]    0    0    0    0    0    0    0    0    0     0     0     0     0     0
[2,]    0    0    0    0    0    0    0    0    1     1     1     1     1     1
[3,]    0    0    0    0    1    1    1    1    0     0     0     0     1     1
[4,]    0    0    1    1    0    0    1    1    0     0     1     1     0     0
[5,]    0    1    0    1    0    1    0    1    0     1     0     1     0     1
     [,15] [,16] [,17] [,18] [,19] [,20] [,21] [,22] [,23] [,24] [,25] [,26]
[1,]     0     1     1     1     1     1     1     1     1     1     1     1
[2,]     1     0     0     0     0     0     0     0     1     1     1     1
[3,]     1     0     0     0     0     1     1     1     0     0     0     1
[4,]     1     0     0     1     1     0     0     1     0     0     1     0
[5,]     0     0     1     0     1     0     1     0     0     1     0     0
```

3.4 Multisets specified by the sum of their elements

Generate all multisets M_l with elements $a_i \in \mathbb{N}$, for which the sum of their elements equals s. Cardinality of these multisets will range from 1 (a multiset $\{s\}$) to s (all elements equal to 1). This problem can be solved with a version of the algorithm that uses weights (Section 2.2). Table 4 illustrates the case with $s = 6$: on the left, there are exact multisets we want to get, on the right - their multiplicities along with the weights for each position, so that the sum of the elements is obtained by summing up products of multiplicities and their corresponding weights ($\sum_{i=1}^{n} m_i w_i$). Since in this case multisets need to satisfy a condition $\sum_{i=1}^{n} m_i w_i = W_{max}$ (and not $\sum_{i=1}^{n} m_i w_i \leqslant W_{max}$ as in Algorithm 2), we run the algorithm without the last position (represented by the rightmost column in Table 4), similarly to the Example 3.1, but in this case keeping the original weights (so that $w_1 = k$; ... ; $w_{k-1} = 2$). The last position is consequently added to bring the sum up to s.

multiset M_l	position weights					
	**6**	**5**	**4**	**3**	**2**	**1**
	multiplicities of M_l					
$\{1, 1, 1, 1, 1, 1\}$	0	0	0	0	0	6
$\{2, 1, 1, 1, 1\}$	0	0	0	0	1	4
$\{2, 2, 1, 1\}$	0	0	0	0	2	2
$\{2, 2, 2\}$	0	0	0	0	3	0
$\{3, 1, 1, 1\}$	0	0	0	1	0	3
$\{3, 2, 1\}$	0	0	0	1	1	1
$\{3, 3\}$	0	0	0	2	0	0
$\{4, 1, 1\}$	0	0	1	0	0	2
$\{4, 2\}$	0	0	1	0	1	0
$\{5, 1\}$	0	1	0	0	0	1
$\{6\}$	1	0	0	0	0	0

Table 4: Multisets, for which the sum of their elements is 6.

Here we generate multisets for $W_{max} = 8$:

```
> # The output is represented by the right side of Table 4
> # (each column in the matrix corresponds to a row in the Table).
> s <- 8
> # Function mirsaW() runs the weighted version of the algorithm
> # eq = TRUE specifies that the sum of the elements should be equal to s
> mirsaW(s, eq = TRUE)

     [,1] [,2] [,3] [,4] [,5] [,6] [,7] [,8] [,9] [,10] [,11] [,12] [,13] [,14]
[1,]    0    0    0    0    0    0    0    0    0     0     0     0     0     0
[2,]    0    0    0    0    0    0    0    0    0     0     0     0     0     0
[3,]    0    0    0    0    0    0    0    0    0     0     0     0     0     0
[4,]    0    0    0    0    0    0    0    0    0     0     0     0     0     0
[5,]    0    0    0    0    0    0    0    0    0     0     1     1     1     1
[6,]    0    0    0    0    0    1    1    1    2     2     0     0     0     1
[7,]    0    1    2    3    4    0    1    2    0     1     0     1     2     0
[8,]    8    6    4    2    0    5    3    1    2     0     4     2     0     1
     [,15] [,16] [,17] [,18] [,19] [,20] [,21] [,22]
[1,]     0     0     0     0     0     0     0     1
[2,]     0     0     0     0     0     0     1     0
[3,]     0     0     0     0     1     1     0     0
[4,]     0     1     1     1     0     0     0     0
[5,]     2     0     0     0     0     0     0     0
[6,]     0     0     0     1     0     0     0     0
[7,]     0     0     1     0     0     1     0     0
[8,]     0     3     1     0     2     0     1     0
```

4 Conclusion

We showed just a few examples to demonstrate the utility and efficiency of the mixed radix incrementing approach. The algorithm is used to solve combinatorial problems that arise in Dcifer likelihood calculation, illustrated by the first three examples. Example 3.1 represents all the combinations of non-unique alleles that are compatible with a given COI and detected (unique) alleles; example 3.2 traverses subcollections of non-unique alleles that are shared between two infections at a given locus (the number of subcollection's elements constrained by M, the number of strain pairs that can be related); and 3.3 represents possible $(IBD_{1,t}, \ldots, IBD_{M,t})$ sequences, where the number of 1's cannot exceed the number of shared non-unique alleles at a locus t. The problem in 3.4 appears, for example, in calculation of higher-order statistics. When a problem can be framed in terms of generating all the possible multisets with some prescribed conditions, it may be solved with this unified approach and algorithm versions tailored to accommodate various kinds of constraints.

References

Donald E Knuth. The Art of Computer Programming, Volume 4A: Combinatorial Algorithms, Part 1. Pearson Education India, 2011.
